# A Comparison between Characterization and Biological Properties of Brazilian Fresh and Aged Propolis

**DOI:** 10.1155/2014/257617

**Published:** 2014-11-03

**Authors:** Eduardo Morgado Schmidt, Daniele Stock, Fabio José Garcia Chada, Daiane Finger, Alexandra Christine Helena Frankland Sawaya, Marcos Nogueira Eberlin, Maria Lurdes Felsner, Sueli Pércio Quináia, Marta Chagas Monteiro, Yohandra Reyes Torres

**Affiliations:** ^1^Universidade Estadual do Centro Oeste (UNICENTRO), CEDETEG, Rua Simeão Camargo Varela de Sá, 03 Vila Carli, 85040-080 Guarapuava, PR, Brazil; ^2^Laboratório Thomson de Espectrometria de Massas, Departamento de Química Analítica, Instituto de Química, Universidade Estadual de Campinas (UNICAMP), 13083-970 Campinas, SP, Brazil; ^3^Programa de Pós-Graduação em Ciências Farmacêuticas, Faculdade de Farmácia, Universidade Federal do Pará (UFPA), Avenida Augusto Correa No. 1, 66075-110 Belém, PA, Brazil; ^4^Departamento de Fisiologia Vegetal, Instituto de Biologia, Universidade Estadual de Campinas, 13083-970 Campinas, SP, Brazil

## Abstract

*Objective*. As propolis is a highly valued bee product, we aimed to verify the quality of aged propolis, investigating their phenolic and flavonoid composition, levels of toxic metals, radical scavenging and antimicrobial activities. *Material and Methods*. Samples of fresh and aged propolis of six different beekeepers, from the same geographical location, were investigated in terms of their phenolic and flavonoid composition and levels of Pb, Cd, and Cr, as well as radical scavenging and antimicrobial activities. *Results*. The two groups of propolis had similar qualitative composition by HPLC-PDA and ESI(-)-MS. Fresh propolis and aged propolis show no differences when average values of extraction yield, flavonoids, EC_50_, or MIC were compared and both types of propolis showed good antimicrobial activity at low concentrations. Only levels of phenolic compounds were higher in fresh propolis. *Conclusion*. The propolis samples considered in this study, aged or fresh, had similar qualitative composition, although they were collected in different periods. Samples only differed in their levels of total phenolic content. Moreover, aged propolis conserves significant radical scavenging and antimicrobial properties. We suggest that aged propolis should not be discarded but explored for alternative applications.

## 1. Introduction

Propolis is a resinous hive substance containing beeswax, plant exudates, and salivary secretions from bees. Extracts of propolis are used as phytochemical ingredient in functional foods at levels that may confer health benefits [1, 2]. The smell, color, constitution, and composition of propolis greatly vary as a function of the different botanical sources available around the hive and the geographical and climatic conditions [3, 4] but also depend on the method of harvest [5].

Flavonoids and phenolic acids or their esters often form up to 50% of all propolis constituents [6]. Several biological activities, such as antibacterial [7, 8], antiviral [7, 9], antioxidant [10, 11], anti-inflammatory [12, 13], anticancer [14–16], and antifungal [17, 18] properties, have been reported for propolis and as a result this resin is a highly valued bee product.

Brazilian propolis is appreciated worldwide. From 2010 to 2012 the price of a kilogram of raw Brazilian propolis increased more than 50% in the international market. According to data from the Japan Trade Organization, 92% of raw propolis consumed in Japan is from Brazil. Propolis which remains long periods in hives (aged propolis) has a dry consistency and is usually discarded by beekeepers. In this study we aimed to verify the quality of aged propolis. Therefore, samples of fresh and aged propolis from six different beekeepers but from the same geographical location were investigated in terms of their phenolic and flavonoid composition and levels of metals, as well as radical scavenging and antimicrobial activities.

## 2. Materials and Methods

### 2.1. Propolis Samples

**Figure 1:**
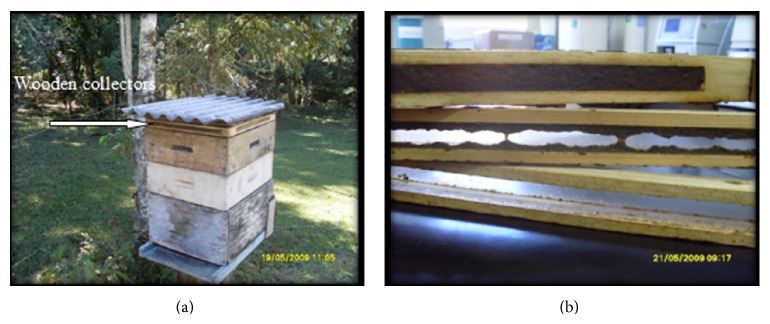
Wooden collectors of propolis (a) placed in the hive to collect fresh propolis and (b) in the lab after 42 days sealed with fresh propolis.

Propolis samples from honeybees (*Apis mellifera*) were collected from hives of six different beekeepers from Prudentópolis, Paraná State, Brazil. On April 7th, 2009, the propolis samples considered as aged (M) (because they had remained in hives for at least 180 days) were collected (1st collection). By the occasion of this collection, wooden collectors were placed in hives to promote the production of fresh propolis (F) (Figure 1). The set of propolis samples considered as fresh propolis were gathered from the wooden collectors on May 19th, 2009 (2nd collection), 42 days after the first collection.

### 2.2. Extracts

Ground propolis (5 g) from both the 1st and 2nd collection was extracted with 50 mL of a 70% v/v hydroalcoholic solution. After stirring in shaker at room temperature for 100 h, these solutions were filtered. The ethanolic solvent was removed under reduced pressure yielding ethanolic extracts of propolis identified as aged propolis M (1st collection) and fresh propolis F (2nd collection).

### 2.3. HPLC Analysis with a Photodiode Array Detector (HPLC-PDA)

The chromatographic profiles of the different propolis extracts were determined by HPLC (Waters 600) with photodiode array detector operating in a 1D detection mode at 292 nm. A thermostatized (30 ± 1°C) *μ*Bondapak C-18 analytical column (3.9 × 300 mm, 10 *μ*m) was used. A binary mobile phase of acetonitrile and 0.5% of aqueous formic acid was employed at an elution rate of 1 mL min^−1^. Linear gradient was performed starting with 30% of acetonitrile to 100% for 30 min. All propolis extract solutions were prepared in acetonitrile at 1000 *μ*g mL^−1^ and 5 *μ*L and were injected through a loop system.

### 2.4. ESI(-)-MS Fingerprints and LC-MS Analysis

Firstly, propolis extracts were analyzed by ESI(-)*-*MS to obtain representative fingerprints and compare their qualitative profiles [19]. Analyses were carried out in a Q-Trap Mass Spectrometer (Applied Biosystems) with direct infusion into the electrospray ionization interface operating in the negative ion mode. Capillary and cone voltages were set to −3000 V and −50 V, respectively. Nitrogen was used as nebulizing and desolvation gas. Desolvation temperature was 100°C.

Subsequently, extracts were introduced into an HPLC (Agillent) with a Waters *μ*Bondapak C18 analytical column (3.9 × 300 mm, 10 *μ*m) and detected in a Triple Quadruple API-5000 mass analyzer. Electrospray ionization was carried out with capillary and cone voltages set to −4000 V and −70 V, respectively. Desolvation temperature was 150°C and nitrogen was used as collision gas. A binary mobile phase of methanol and 1% of aqueous formic acid was employed at an elution rate of 1 mL min^−1^. Elution started with 40% of methanol in 1% of aqueous formic acid for 25 min. After that period, a linear gradient was performed for 30 min until 100% methanol. The chromatographic system was allowed to equilibrate for 5 min between injections.

### 2.5. Total Phenolic Content

The amount of total phenolic components was determined by the Folin-Ciocalteau method with some modifications [15, 20, 21]. Solutions of propolis extracts were prepared in methanol at a concentration of 1000 *μ*g mL^−1^. In a 5 mL volumetric flask, 500 *μ*L of a buffer solution (20 g sodium carbonate and 1.2 g sodium potassium tartrate in 100 mL of water), 500 *μ*L of Folin-Ciocalteau reagent (Biotec, 2 mol L^−1^), and 300 *μ*L of the analytical standard or propolis extract solution were mixed and the volume was completed with ultrapure water (PKA Genpure). Absorbance was measured in a Varian Cary 50 Bio UV-Vis spectrophotometer at 760 nm after 30 min at room temperature. The calibration curve was set up by measuring the absorbance of the commercial gallic acid (Vetec, 99%) standard solutions ranging from 10 to 280 *μ*g mL^−1^. The total phenolic content was expressed in mg of gallic acid per g of propolis extract.

### 2.6. Flavonoid Content

The amount of total flavonoids was determined by the method that employs dihydrate aluminum chloride in methanol [15, 21]. Solutions containing propolis extracts at 1000 *μ*g mL^−1^ were prepared in methanol. In a 5 mL volumetric flask, 500 *μ*L of each analytical solution (standard or propolis extracts) and 250 *μ*L of aluminum chloride methanolic solution (5% w/v) were mixed and diluted with methanol. After 30 min absorbance was measured at 425 nm in a Varian Cary 50 Bio UV-Vis spectrophotometer. Quercetin (Sigma, 98%) was employed as analytical standard in concentrations ranging from 1 to 50 *μ*g mL^−1^ and the results were expressed as mg of quercetin per g of propolis extract.

### 2.7. Determination of Levels of Metals

Levels of Cd, Cr, and Pb were evaluated in propolis extracts by atomic absorption spectrophotometry (FAAS). A Varian AA-220 atomic absorption spectrometer equipped with a deuterium-arc lamp background corrector was used. Cd, Cr, and Pb were analyzed in an air-acetylene flame. Samples of 0.025 g of each propolis extract (in triplicate) were dissolved in methanol in a 25 mL volumetric flask. Aliquots from these solutions were directly aspirated into the FAAS. The same procedure was performed with metal standard solutions in methanol. The burner height and the flow rates of sample and acetylene were adjusted in order to obtain the maximum absorbance signal.

### 2.8. Radical Scavenging Activity

The radical scavenging activity was determined by the DPPH test. A stock solution of DPPH (1.6 × 10^−3 ^mol L^−1^) was made in ethanol and filtered through Milli Q. The working DPPH ethanolic solutions (8.0 × 10^−5 ^mol L^−1^) were prepared directly in a plastic cuvette for every measurement. All solutions of propolis extracts were prepared in ethanol at a concentration of 1000 *μ*g mL^−1^ and different aliquots were removed from these solutions to construct the analytical curve. The mixture of DPPH and antioxidants in increasing concentrations was left to stand for 30 min at room temperature in the dark and then absorbance was measured at 515 nm. Antiradical activity of the extracts was expressed as EC_50_, meaning the concentration of propolis extract that reduced in 50% the absorbance of the working DPPH ethanolic solutions at the initial concentration of 8.0 × 10^−5 ^mol  L^−1^. To calculate EC_50_ an analytical curve for antiradical activity (%) versus extract concentration (*μ*g mL^−1^) was plotted. The radical scavenging activity was calculated according to the following formula:

(1) $$\begin{array}{c} \%\;\text{Antiradical}\;\text{activity}=100\times \frac{\left({\text{Abs}}_{i}-{\text{Abs}}_{f}\right)}{{\text{Abs}}_{i}}, \end{array}$$

where Abs_*i*_ is absorbance of working DPPH ethanolic solutions, *t* = 0, and Abs_*f*_ is absorbance of DPPH ethanolic solutions containing different concentrations of antioxidants, *t* = 30 min.

### 2.9. *In Vitro* Antimicrobial Activity

Antibacterial activity was evaluated for the following standard strains: (i) Gram-positive bacteria,* Staphylococcus aureus* (ATCC 6538),* Enterococcus faecalis* (ATCC 29212), and* Micrococcus luteus* (ATCC), and (ii) Gram-negative bacteria,* Pseudomonas aeruginosa* (ATCC 25853) and* Escherichia coli* (ATCC 8739). All strains were obtained from the INCQS/FIOCRUZ (National Institute for Health Quality Control, Brazil). The microorganisms used in the study were maintained in the Laboratory of Microbiology at College of Pharmacy, Federal University of Pará, UFPA. The standard strains were kept in nutrient agar at room temperature. For the tests, all strains were grown in Petri dishes containing a specific media for each bacterium: mannitol salt agar medium to grow* S. aureus*; nutrient agar for* E. faecalis*; cetrimide agar for* P. aeruginosa*, and MacConkey Agar for* E. coli*. Plates were incubated at 37°C for 24 h to induce the exponential growth after lag time.

For bacterial inoculum preparation, strains were grown to exponential phase in Mueller-Hinton broth (Merck, Germany) at 37°C for 24 h and adjusted by diluting fresh cultures to turbidity equivalent to 0.5 McFarland scale (approximately 2 × 10^8^ CFU mL^−1^) and then diluted until 1 × 10^3^ CFU mL^−1^, as described by Clinical and Laboratory Standards Institute [22].

Minimum inhibitory concentration (MIC) and minimum bactericidal concentration (MBC) assays were performed by using the broth microdilution method in Mueller-Hinton broth (MHB) as described by CLSI [22]. MIC is defined as the lowest concentration of extract with no visible growth of the microorganism in the resazurin colorimetric assay. To determine MIC, fresh and aged propolis extract were dissolved in dimethylsulfoxide (DMSO) in the highest concentration (8000 *μ*g mL^−1^) to be tested. A serial twofold dilution was made in a concentration range from 100 to 8000 *μ*g mL^−1^ in 1 mL sterile test tubes containing MHB.

For the microdilution test, the inoculum (100 *μ*L) containing 5* ×* 10^3^ CFU mL^−1^ was added to each well and 100 *μ*L from their serial dilutions was transferred into consecutive wells. After 24 h of incubation, 15 *μ*L of resazurin (1 *μ*g mL^−1^), which is metabolically reduced by active cells to a colored derivative, was added to the wells to allow visual identification of metabolic activity [23]. After incubation, the development of a purple-pink color was considered as the indicative of bacterial growth. Therefore MIC was read as the lowest concentration of the extract where the purple-pink color was not observed. To determine MBC, 10 *μ*L of broth was taken from each well and incubated in Mueller Hinton Agar at 37°C for 24 h and for each bacterium. The MBC was defined as the lowest extract concentration that resulted in a colony count lower than three colonies per mL (99.9% killing) or no bacterial growth, as described by de Quadros et al. [24]. Each test was performed in three replicates. Negative control consisted of 100 *μ*L of the bacterial inoculum and 100 *μ*L of DMSO. Chloramphenicol (250 *μ*g mL^−1^) and penicillin/streptomycin (100 U mL^−1^) were used as positive controls for Gram-positive and Gram-negative bacteria, respectively.

### 2.10. Statistical Analysis

The differences between average values of extraction yield, phenolic and flavonoid amounts, and also antiradical and antimicrobial activities, for aged and fresh propolis (two experimental groups), were investigated by a one-way ANOVA at 95% confidence level. To identify the average values which statistically differ from each other, a Tukey multiple comparisons mean test, at the same confidence level, was applied.

## 3. Results

### 3.1. Comparison of the Qualitative Profiles of Fresh and Aged Propolis Samples

**Figure 2:**
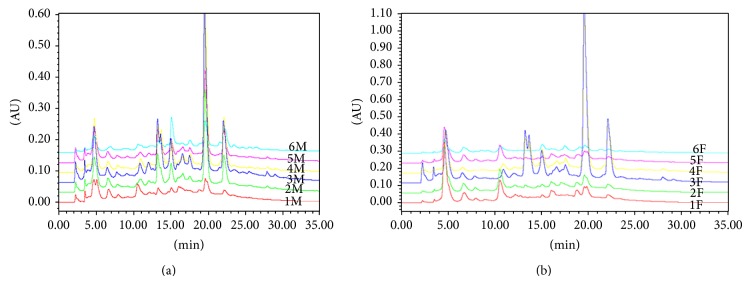
Chromatograms detected at 292 nm for propolis extracts from different beekeepers. (a) Aged propolis and (b) fresh propolis.

According to beekeepers who provided samples for the current study, propolis from the 1st collection had not been harvested for at least 180 days and had a drier consistency and darker color than the fresh propolis samples. The 2nd collection was carried out 42 days after the 1st collection. All propolis samples were extracted with ethanol 70% (v/v). An aliquot of each extract was injected in HPLC and analyzed by HPLC-PDA and ESI(-)*-*MS. The analyses by HPLC*-*PDA and chromatograms detected at 292 nm showed that although propolis samples were obtained from different beekeepers and different periods (but from nearby areas) their chromatographic profiles were similar (Figure 2). However, propolis 3 and propolis 4 had higher concentration of major components, especially the peaks with *t*_*R*_ 20 and 22 minutes.

**Figure 3:**
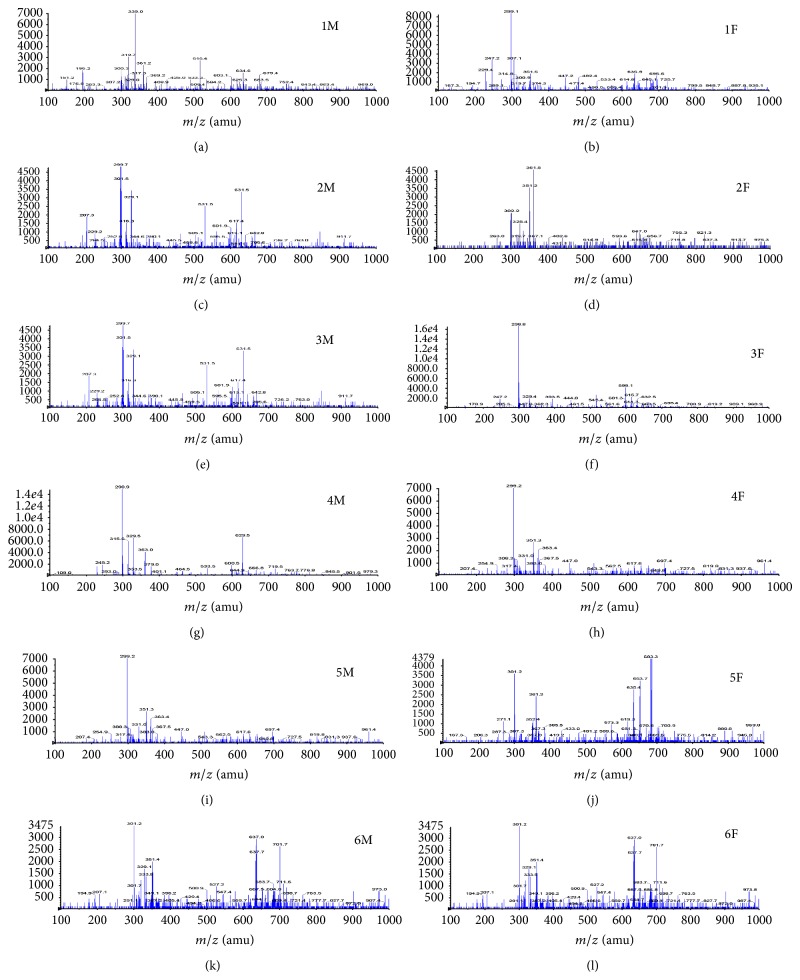
ESI(-)-MS fingerprints for aged (M) and fresh (F) propolis extracts from different beekeepers.

ESI(-)*-*MS fingerprints showed a complex chemical composition and ions of* m/z* 299 or* m/z* 301 were observed in the fingerprints of all samples (Figure 3). LC*-*MS analysis allowed the identification of several compounds which we have reported earlier in our studies with propolis from Prudentópolis (Paraná) [15, 16]. Dicaffeoylquinic acid isomers with [M–H]^−^ ions of* m/z* 515 but different retention times were detected. Ions of* m/z* 163 (*t*_*R*_ 7.2),* m/z* 231 (*t*_*R*_ 16.5), and* m/z* 329 (*t*_*R*_ 19.8) were identified as the [M–H]^−^ ions of p-coumaric acid, 4-hydroxy-3-prenylcinnamic acid, and betuletol, respectively. Ions of* m/z* 299 were detected at two different retention times (17.2 and 21.6) and were attributed to kaempferide and 3,5-diprenyl-4-hydroxycinnamic acid (artepillin C). Similarly, ions of* m/z* 301 (at 12.8 min and 25.5 min) were attributed to dihydrokaempferide and* E/Z* communic acid, respectively.

### 3.2. Total Phenolic and Flavonoid Contents in Fresh and Aged Propolis

When extraction yield, total phenolic and flavonoid levels for aged and fresh propolis were compared in pairs for the same beekeeper, a trend toward higher amounts of extraction yield and total phenolic acids in fresh propolis is noticed (Table 1). Conversely, the amount of flavonoids was slightly superior in aged propolis, with the exception of propolis from beekeepers 3 and 4 for which no statistical differences were observed.

### 3.3. Radical Scavenging Activity by the DPPH Assay

The DPPH assay was performed for aged and fresh propolis obtained from different beekeepers in order to verify the effect that long periods in hive have on propolis radical scavenging activity (Table 1). All extracts obtained from fresh propolis had higher radical scavenging activity (lower EC_50_ value) than the extracts obtained from aged propolis from the same beekeeper, except for extracts 6M and 6F.

### 3.4. Antimicrobial Activity

The* in vitro* antibacterial activity was assessed through the values of MIC and MBC of six aged (M) and six fresh (F) propolis samples against strains of Gram-positive and Gram-negative bacteria.  Table 2 shows that all extracts, regardless of being from aged or from fresh propolis, were able to inhibit the growth of Gram-positive bacteria, mainly* S. aureus* and* M. luteus*, at low concentrations showing a good antimicrobial activity. These propolis extracts were however not effective against Gram-negative bacteria, with MIC values higher than 4000 *μ*g mL^−1^ (data not shown).

### 3.5. Statistical Analysis

To statistically compare aged and fresh propolis samples, they were considered as two experimental groups and the quantitative data obtained were analyzed by a one-way ANOVA. No statistical difference at the 95% confidence level was observed between average values of (i) extraction yield (aged propolis: 64 ± 8%, fresh propolis: 71 ± 11%); (ii) flavonoids levels (aged propolis: 14 ± 4 mg g^−1^, fresh propolis: 12 ± 6 mg g^−1^), and (iii) EC_50_ in DPPH test (aged propolis: 47 ± 15 *μ*g mL^−1^, fresh propolis: 43 ± 22 *μ*g mL^−1^), considering the six beekeepers altogether. The same result was obtained when MIC and MBC mean values for each bacterium were statistically compared: (i)* Staphylococcus aureus* (aged propolis: MIC 650 ± 353 *μ*g mL^−1^ and MBC 1015 ± 777 *μ*g mL^−1^, fresh propolis: MIC 382 ± 13 *μ*g mL^−1^ and MBC 765 ± 455 *μ*g mL^−1^); (ii)* Enterococcus faecalis* (aged propolis: MIC 1822 ± 656 *μ*g mL^−1^ and MBC 3643 ± 1324 *μ*g mL^−1^, fresh propolis: MIC 1352 ± 767 *μ*g mL^−1^ and MBC 2972 ± 1427 *μ*g mL^−1^);* Micrococcus luteus* (aged propolis: MIC 400 ± 124 *μ*g mL^−1^ and MBC 1040 ± 404 *μ*g/mL, fresh propolis: MIC 435 ± 120 *μ*g mL^−1^ and MBC 935 ± 432 *μ*g mL^−1^). For Gram-negative strains, the antibacterial activity was weak for both fresh and aged propolis. Extracts of propolis are extensively described as being effective against Gram-positive bacteria but very weak against Gram-negative ones [15, 25]. Aged and fresh propolis had however mean phenolic values which statistically differ at the 95% confidence level with a trend to fresh propolis showing higher levels (133 ± 18 mg g^−1^) than the aged samples (107 ± 10 mg g^−1^).

## 4. Discussions

The main purpose of the current study was to verify if propolis which remains for long period in hives is of lower quality, considering important parameters such as total phenolic and flavonoid contents, radical scavenging and antimicrobial activities, and levels of toxic metals. Aged propolis was compared to fresh propolis from the same geographical area.

The qualitative chemical profile observed by HPLC-PDA, ESI(-)-MS and LC-MS for all samples was alike despite aged and fresh propolis having been collected in different periods and from different beekeepers (Figures 2 and 3). Consequently, it was assumed that the qualitative composition of these propolis samples was not significantly affected by seasonal effects. As aged and fresh propolis had similar ions in their fingerprint it can be deduced that bees used the same plant sources to collect the resin. The vegetal origin of propolis from Paraná State is complex and* Baccharis dracunculifolia, Araucaria heterophylla and Araucaria angustifolia *have been suggested as possible plant sources [19, 26, 27]. Park et al. [28] reported based on physicochemical characteristics that the main botanical origin of propolis from southeastern Brazil was* Baccharis dracunculifolia* DC. (Compositae), popularly known as “alecrim-do-campo,” which is largely distributed in South America from southeastern Brazil to Argentina and Uruguay. Propolis from* B. dracunculifolia* is rich in phenolic acids, particularly prenylated derivatives of p-coumaric acid, as shown in our data.

Among tropical countries, Brazil has the widest chemical diversity of propolis types; however variations in qualitative chemical composition of Brazilian propolis due to seasonal effect are not always observed. In this regard, Simões-Ambrosio et al. [29] evaluated the role of seasonality on the inhibitory effect on the oxidative metabolism of neutrophils of Brazilian green propolis collected monthly from November 2001 to October 2002. The authors verified that the HPLC qualitative profiles of the extracts were very similar. Nonetheless, there was wide variation in the quantitative profile which resulted in significant differences in the inhibitory effects of the propolis samples during the studied period. The same way, Teixeira et al. [30] observed that most compounds of a sample of Brazilian propolis (from Minas Gerais State) were detected throughout a year but their contents varied along the year. Additionally, the lack of seasonal effects on the antimicrobial activity against* Staphylococcus aureus* and* Escherichia coli *[25] and against* Candida albicans* [17, 18] and on the immunomodulatory action [31] of propolis collected from the same geographical region in São Paulo State, Brazil, in four seasons throughout a whole year was reported.

The amounts of phenolic and flavonoid constituents vary widely according to propolis types and seasonal factors [30, 32, 33]. Moreover, some studies reported that the phenolic content is related to the various pharmacological activities reported for propolis, such as antibacterial, anti-inflammatory, hepatoprotective, and antioxidant activities. [34, 35]. In this study, the only variable which statistically differed between aged and fresh propolis was the total phenolic content; however these differences in content did not reduce significantly the radical scavenging and antimicrobial activities of the aged propolis. Several studies showed that propolis from tropical regions contains a diversity of phenolics, such as prenylated cinnamic acid derivatives, flavonoids, polyprenylated benzophenones, and other classes of constituents [7, 18, 36]. In a previous study, we identified several prenylated phenolic acids in an extract of propolis obtained with edible vegetable oil (ODEP) such as 3,4-dihydroxy-5-prenyl-cinnamic acid, 3-prenyl-4-hydroxycinnamic acid, and (E)-3-{4-hydroxy-3-[(E)-4-(2,3-dihydrocinnamoy-loxy)-3-methyl-2-butenyl]-5-prenyl-phenyl}-2-propenoic acid [18]. This oil extract was obtained from a propolis sample collected in Prudentopolis, Paraná State (the same area as the samples in this study). ODEP showed antifungal activity against several strains of* Candida albicans* [18]. Recently, we also reported that this extract exerted hyperlocomotor and anxiolytic- and antidepressant-like effects in the CNS in different animal models, as well as antioxidant activity after stress induced by the forced swim test [37].

The potent antioxidant activity of Brazilian propolis observed in our study was also shown by Guimarães et al. [38] who tested propolis collected from Brazil with strong DPPH free radical scavenging activity (ED50 values around 45.43 *μ*g/mL). In this regard, Banskota et al. [39] reported that the antioxidative activity of propolis is due to its phenolic constituents, which also possess antitumour and antihepatotoxic activities. These compounds may reduce intracellular peroxides levels, such as ROS, which acts as second messengers for signal transduction pathways that regulate cell proliferation and are associated with tumour promotion and induction of the carcinogenesis.

Concerning the antimicrobial activity, when fresh and aged propolis are pair-compared for the same beekeeper slightly better radical scavenging and antimicrobial activities were found for fresh propolis which showed a good antimicrobial activity mainly against* S. aureus*. Nevertheless, when these activities are compared considering all the fresh samples and all the aged samples, no statistical differences between average values were observed. Others studies also reported that propolis is active mainly against Gram-positive bacteria but shows a limited activity against Gram-negative bacteria [40–43]. These variations in the susceptibility to propolis among several microorganisms have been reported, but their mechanisms of action are poorly disclosed. Then, the mechanism of antimicrobial activity of propolis is complex and is attributed to a synergism between phenolic and other compounds in the resin [44]. Though, some studies suggested that propolis and some of its components had an antibacterial effect due to damage to the cytoplasmic membrane of bacteria and so increasing its permeability providing the leakage of the important intracellular solute potassium [39, 45–47]. In addition, Mirzoeva et al. [45] reported that propolis and some of its components altered ionic permeability of the bacterial inner membrane leading to dissipation of the membrane potential due to the electrochemical gradient of protons across the membrane. This is essential to maintain ATP synthesis, membrane transport, and motility of the bacteria. In this regard, Cushnie and Lamb [46] showed that galangin, an important component of propolis, may induce potassium loss producing damage on cytoplasmic membrane, weakening the cell wall, or the inhibition of its synthesis and thereby resulting in osmotic lysis.

Finally, it is also important to highlight that toxic elements such as Pb, Cd, and Cr were not detected in fresh or aged propolis extracts. The presence of these metals in propolis has been attributed to environmental contamination of air, plants, soil, or waters around the hives due to anthropic activities [48]. Pb, Cd, and Cr were below detection limits in extracts from aged and fresh propolis despite the fact that aged propolis remained more time in hives and were accumulated in hives during a more extended period than fresh propolis, being more exposed to environmental conditions. In Brazil, the Ministry of Agriculture, Livestock and Supply—MAPA—approved a technical protocol to define the identity of bee products and minimal parameters for their quality control [49]. In this protocol a minimum amount of 0.25% (w/w) of flavonoids and 0.50% (w/w) for phenolics in extracts of propolis extracts is established. It also states that inorganic contaminants must not be present in propolis or extracts in higher amounts than those defined by the specific regulation for honeys. Currently, MAPA has implemented “The National Plan for Control of Residues and Contaminants in Products of Animal Origin” (PNCRC/ANIMAL) as a tool to ensure quality throughout the productive chains. The tolerable limit concentration for Cd and Pb in honey is 100 and 500 ng g^−1^, respectively [50] and for Cr is 100 ng g^−1^ [51]. We can conclude that extracts from aged and fresh propolis meet the technical protocols established by the Brazilian legislation regarding the content of inorganic contaminants in products of animal origin as well as phenolics and flavonoids levels in extracts of propolis.

## 5. Conclusions

This study showed that despite being collected in different periods, propolis samples had a very similar qualitative composition. Samples differed from each other only in relation to their levels of total phenolic content (93.67–149.30) mg/g. Even though aged propolis generally has a different appearance and a drier consistency and, according to beekeepers, this type of propolis is depreciated, data collected in this study indicates that aged propolis still has significant radical scavenging and good antimicrobial activities. These results therefore suggest that aged propolis should not be discarded. Toxic metals (Pb, Cr, and Cd) were not detected in propolis extracts.

## Figures and Tables

**Table 1:** Total phenolic, flavonoids, yields, and radical scavenging activity for extracts of aged (M) and fresh (F) propolis from beekeepers 1 to 6^*^.

Propolis	Yield (%)	Total phenolic content (mg g^−1^)	Total flavonoid content (mg g^−1^)	DPPH EC_50_ (*µ*g mL^−1^)
1M	67	108.9 ± 3.2	9.3 ± 0.1	49.88
1F	81	149.3 ± 5.1	6.7 ± 0.4	36.60
2M	65	114.8 ± 5.4	14.0 ± 0.4	38.50
2F	85	147.4 ± 4.1	11.1 ± 0.5	35.65
3M	64	120.5 ± 3.6	21.0 ± 0.3	27.52
3F	65	143.9 ± 3.0	20.9 ± 0.9	17.13
4M	77	106.7 ± 2.6	16.5 ± 1.2	43.59
4F	70	121.8 ± 0.8	17.7 ± 0.6	36.52
5M	53	99.9 ± 1.8	14.1 ± 2.5	50.83
5F	60	131.3 ± 0.6	9.1 ± 0.2	48.67
6M	56	93.7 ± 0.2	10.6 ± 0.3	73.26
6F	62	101.9 ± 4.2	6.0 ± 0.2	83.60

M	64 ± 8	107 ± 10^a^	14 ± 4	47 ± 15
F	71 ± 11	133 ± 18^b^	12 ± 6	43 ± 22

^*^Data are represented as average values ± standard deviation. Different letter represents significant statistical differences (*P* < 0.05) between average values for aged and fresh propolis.

**Table 2:** Minimum inhibitory concentration (MIC) and minimum bactericidal concentration (MBC) for extracts of aged (M) and fresh (F) propolis from beekeepers 1 to 6.

Propolis	Bacteria—MIC and MBC (*μ*g mL^−1^)					
	*Staphylococcus aureus *		*Enterococcus faecalis *		*Micrococcus luteus *	
	MIC	MBC	MIC	MBC	MIC	MBC
1M	1300	2600	2600	>5190	650	1300
1F	400	1620	810	1620	400	810
2M	560	780	1560	3110	390	1560
2F	360	680	2730	5450	680	1360
3M	340	680	2720	2720	340	680
3F	380	380	1500	1500	380	750
4M	340	670	1340	2680	340	1340
4F	380	380	770	3100	380	770
5M	690	690	1370	5480	340	690
5F	390	770	1540	>3090	390	1540
6M	670	670	1340	2680	340	670
6F	380	760	760	3070	380	380

M	650 ± 353	1015 ± 777	1822 ± 621	3643 ± 1324	400 ± 124	1040 ± 404
F	382^*^ ± 13	765 ± 455	1352 ± 767	2972 ± 1427	435 ± 120	935 ± 432

^*^Data are represented as average values ± standard deviation (*P* < 0.05).
